# Late positive complex in event-related potentials tracks memory signals when they are decision relevant

**DOI:** 10.1038/s41598-019-45880-y

**Published:** 2019-07-01

**Authors:** Haopei Yang, Geoffrey Laforge, Bobby Stojanoski, Emily S. Nichols, Ken McRae, Stefan Köhler

**Affiliations:** 1 Brain and Mind Institute, Graduate Program in Neuroscience, London, N6A 3K7 Canada; 2 Department of Psychology, Brain and Mind Institute, London, N6A 3K7 Canada; 3 Brain and Mind Institute, Department of Physics, London, N6A 3K7 Canada; 4 Rotman Research Institute, Baycrest Centre, Toronto, M6A 2X8 Canada

**Keywords:** Cognitive neuroscience, Long-term memory

## Abstract

The Late Positive Complex (LPC) is an Event-Related Potential (ERP) consistently observed in recognition-memory paradigms. In the present study, we investigated whether the LPC tracks the strength of multiple types of memory signals, and whether it does so in a decision dependent manner. For this purpose, we employed judgements of cumulative lifetime exposure to object concepts, and judgements of cumulative recent exposure (i.e., frequency judgements) in a study-test paradigm. A comparison of ERP signatures in relation to degree of prior exposure across the two memory tasks and the study phase revealed that the LPC tracks both types of memory signals, but only when they are relevant to the decision at hand. Another ERP component previously implicated in recognition memory, the FN400, showed a distinct pattern of activity across conditions that differed from the LPC; it tracked only recent exposure in a decision-dependent manner. Another similar ERP component typically linked to conceptual processing in past work, the N400, was sensitive to degree of recent and lifetime exposure, but it did not track them in a decision dependent manner. Finally, source localization analyses pointed to a potential source of the LPC in left ventral lateral parietal cortex, which also showed the decision-dependent effect. The current findings highlight the role of decision making in ERP markers of prior exposure in tasks other than those typically used in studies of recognition memory, and provides an initial link between the LPC and the previously suggested role of ventral lateral parietal cortex in memory judgements.

## Introduction

Recognition memory refers to the ability to recognize that a stimulus has been encountered previously. Due to its broad functional significance in cognition, it has been studied in laboratory settings with a variety of behavioral and neuroimaging methods, including a rich body of literature focusing on event-related potentials (ERPs; for a review see^1^). Most of the studies have employed variants of study-test paradigms that require discriminating items that had been previously encountered in a study phase from non-studied novel stimuli. Two ERP components have been identified that have been shown to track the outcome of participants’ memory judgements in many studies. First, the late positive complex (LPC) is a positive-going ERP that peaks around 600 ms after stimulus onset with a central posterior topography. Stimuli judged as old (i.e., previously encountered in an experimental study phase) typically elicit a more positive LPC than do those judged as new^2^. The second ERP component is the mid-frontal FN400, characterized by an earlier peak at 400 ms, with a more positive deflection for stimuli judged to be old. These two ERP components have often been interpreted in the context of the dual-process model of recognition memory, with the LPC marking recollection of episodic details about the prior stimulus encounter, and the FN400 marking item-based familiarity assessment (devoid of episodic context). This popular interpretation of the two ERP components of recognition memory has, however, been questioned in recent years, and the exact processes that underlie them remain a contentious issue^3,4^. The current research addresses the functional significance of the LPC in light of recent evidence that ties it to aspects of decision making during memory judgements, rather than a unique role in episodic recollection^5,6^. We examine this idea in the context of two memory tasks not frequently employed in prior research, which focus on assessment of different types of cumulative prior exposure, rather than discrimination between previously studied and novel stimuli.

Results from a recent study called into question the classic interpretation of the LPC as a specific marker of episodic recollection by suggesting that the LPC tracks the perceived strength of memory (reflected in confidence) even when participants cannot recollect episodic detail pertaining to the stimulus encounter^5^. Brezis *et al*. (2016) employed a variant of the Remember/Know (RK) procedure, commonly used in recognition-memory experiments to distinguish familiarity from recollection. In a RK paradigm, participants are asked to not only indicate whether they have encountered the stimuli in the study phase, but also to specify whether they can recollect episodic details of that encounter (‘Remember’) or not (‘Know’). They added a component to the memory judgement by asking participants to provide confidence ratings of their old/new responses prior to indicating the basis of recognition of old items by choosing “Remember”, “Know”, or “Guess”. Their core analyses showed that high confidence Know responses elicited a more positive LPC than low confidence Remember responses. This result suggests that, while tied to the outcome of the memory decision, the LPC is not an exclusive marker of episodic recollection. Instead, it may be a broader marker of the strength of the signal that drives the decision as measured by expressed confidence. From this perspective, the LPC may indeed also track other types of memory signals, to the extent that they are relevant to the memory decision.

Evidence that favors an interpretation of the LPC as a marker of signal strength for the memory decision at hand also comes from a recent EEG-based study that fitted a drift-diffusion model to single-trial data from a recognition-memory experiment that required participants to make old/new judgements about items from a list of words^6^. The drift-diffusion model simulates decision-making processes as an accumulation of noisy evidence. Studies using this model typically estimate its parameters using reaction time and accuracy data, and then examine the estimated parameters as markers of the underlying mechanisms of decision making. Ratcliff *et al*. (2016) first trained classifiers to label individual trials as “studied” or “unstudied” based on objective item status, using EEG data from multiple time windows (approximately FN400 or the LPC time windows). They found that only the classifier trained on the LPC time window predicted later behavioral performance on a trial-by-trial basis. Specifically, the drift rate, a parameter that corresponds to the speed of evidence accumulation, differed significantly only when the drift-diffusion model was fitted with the classifier output from this time window. Ratcliff *et al*. concluded that the LPC tracks evidence accumulation, and that it is the only electrophysiological component that contains information that drives memory judgements.

In the studies outlined above, as well as in the ERP literature at large, the memory judgements required discrimination of previously studied from non-studied items. In such a study-test paradigm, participants always make memory judgements with respect to an experimentally controlled study phase. However, when meaningful stimuli such as object concepts (i.e., the concrete object to which a word or picture refers^7,8^) are used, participants also have varying degrees of pre-experimental exposure that can lead to different memory strengths. Indeed, it has been argued that recognition-memory studies conducted with meaningful stimuli and with memory judgements that probe exposure in an experimental study phase tap into the degree of recent change in memory strength rather than absolute cumulative strength^7,9^. Behavioral findings suggest that humans can also judge cumulative past exposure to object concepts accrued over their lifetime outside the laboratory. For example, people can easily judge whether they have had more lifetime exposure to apples or tangerines. Such judgements display considerable consistency across participants within a given culture^10–12^, and engage brain structures in the medial temporal lobe that are known to play a critical role in recognition memory^7,13^.

In the present study, we employed judgements of lifetime exposure to determine whether and how the LPC is tied to decision-making in memory judgements. Specifically, we asked whether the LPC can flexibly track the strength of multiple types of memory signals to the extent that they are decision-relevant. For this purpose, we also used relative frequency judgements that assess cumulative recent item exposure, which was directly manipulated in an experimental study phase with an incidental semantic encoding task. We predicted that if the LPC does indeed track the strength of multiple types of memory signals in a decision-dependent manner, it should be sensitive to the amount of cumulative lifetime exposure when participants are making lifetime exposure judgements, but not when participants are judging the relative frequency of cumulative recent exposure. Similarly, it should be sensitive to the amount of cumulative recent exposure during frequency judgements but not during the semantic judgements in the experimental study phase. Because the LPC has previously been linked to left ventral lateral parietal cortex in intracranial electrocorticography (ECoG) and recordings from depth electrodes in humans^14–16^, we hypothesized that cortical source activity in this region would show a similar effect during the LPC time window. To determine whether these predicted results are specific to the LPC, we also analyzed the FN400/N400 ERP component.

## Results

### Behavioral results

**Figure 1:**
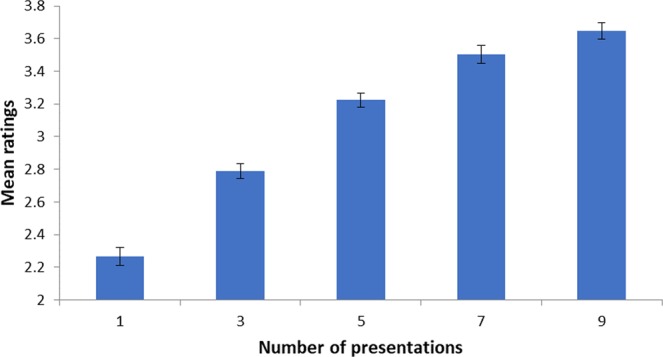
Mean ratings given to each presentation frequency bin in the test phase for recent exposure. Error bars represent standard errors of the means across participants.

**Figure 2:**
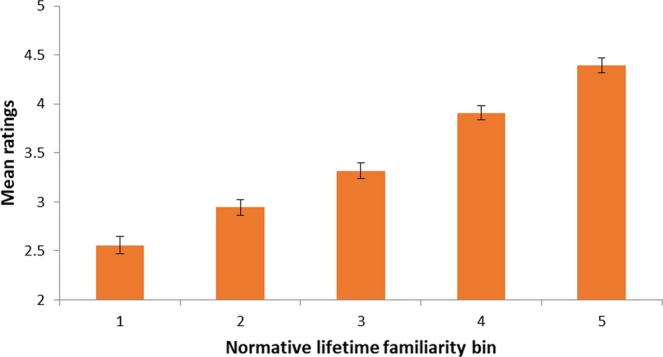
Mean ratings given to each normative lifetime familiarity bin in the test phase. Bins are defined based on normative data reported by^17^. Error bars represent standard errors of the means across participants.

To quantify participants’ memory performance in the recent exposure task, we correlated participants’ judged relative frequency in the test phase with the actual number of repetitions in the study phase. Significant positive correlations (at p < 0.05) were observed in 53 out of 57 participants, with a mean r (123) = 0.41, p < 0.001, indicating sensitivity of these judgements to our exposure manipulation (Fig. 1). To quantify performance in the lifetime exposure task, we followed a procedure employed in our prior work^7^. We correlated participants’ ratings with those reported in a normative database^17^. Significant positive correlations were observed in 54 out of 57 participants, with a mean r (123) = 0.51, p < 0.001, again indicating sensitivity to the memory dimension of interest (Fig. 2).

It is evident that the mean response time (RT) was shorter for the study phase (Table 1) as compared to the test phase (Table 2). However, when comparing the RT differences calculated according to the ERP contrasts (“high” – “low”, see below) using permutation tests, we found that these RT differences related to degree of exposure were closely matched across the two tasks in the test phase, and between study and test phase, all ps > 0.7.

**Table 1:** Response times in milliseconds in the study phase (Means and Standard Errors).

Numbers of presentation									
	1st	2^nd^	3rd	4th	5th	6th	7th	8th	9th
Bin 1	683.78 (9.84)								
Bin 2	683.87 (9.04)	657.09 (10.12)	644.58 (10.08)						
Bin 3	691.04 (10.08)	649.82 (10.68)	649.95 (9.36)	645.54 (11.16)	628.28 (10.25)				
Bin 4	694.66 (9.30)	656.47 (9.10)	643.89 (9.05)	642.03 (10.78)	635.38 (10.85)	628.34 (9.92)	627.33 (11.13)		
Bin 5	691.99 (8.98)	652.53 (10.15)	640.34 (10.34)	639.03 (10.69)	631.95 (9.67)	624.44 (11.40)	627.01 (10.03)	620.77 (10.52)	618.30 (10.65)

**Table 2:** Response times in milliseconds in the test phase (Means and Standard Errors).

Relative frequency judgement					Lifetime exposure judgement				
1	2	3	4	5	1	2	3	4	5
1356.29 (30.79)	1436.70 (30.64)	1397.65 (46.09)	1346.00 (30.47)	1222.37 (30.56)	1324.25 (30.59)	1475.17 (33.91)	1476.00 (51.05)	1437.82 (36.06)	1188.44 (31.37)

### Does the LPC track perceived prior exposure in cumulative memory judgements?

To obtain a sufficient number of trials for critical comparisons along the memory dimensions in the two types of memory judgements, and to maximize stability of the corresponding waveforms, we computed weighted ERP averages for high- versus low-exposure bins. Specifically, responses for the upper most response levels (i.e. 4 and 5) were contrasted with those for the lower most response levels (i.e. 1 and 2). For the recent-exposure task, the high and low combined levels had on average 43 and 38 trials, respectively, corresponding to average rejection rates of 13% for both levels. For the lifetime exposure task, the high and low combined levels had on average 53 and 35 trials, corresponding to average rejection rates of 15% and 13%, respectively. ANOVAs were conducted on the four ROIs (left-right and anterior-centroposterior, see Methods for details). For the LPC time window (500–800 ms), we first conducted an omnibus repeated measures ANOVA on the ERPs with anteriority (2 levels), laterality (2 levels), task (2 levels), and response (2 levels) as factors.

The omnibus test yielded significant main effects of anteriority, F (1, 46) = 49.81, p < 0.001; ${\hat{\eta }}_{G}^{2}$ = 0.14; and laterality, F (1, 46) = 14.28, p < 0.001, ${\hat{\eta }}_{G}^{2}$ = 0.01. Significant two-way interactions were observed for anteriority × response, F (1, 46) = 18.96, p < 0.001, ${\hat{\eta }}_{G}^{2}$ = 0.005; laterality × task, F (1, 46) = 8.97, p = 0.004, ${\hat{\eta }}_{G}^{2}$ < 0.001; and anteriority × laterality, F(1, 46) = 4.04, p = 0.050, ${\hat{\eta }}_{G}^{2}$ < 0.001. All other effects were non-significant, F < 4.04, p > 0.1, ${\hat{\eta }}_{G}^{2}$ < 0.005.

Because our primary interest is in effects related to the memory judgements, we performed simple-effect post hoc tests on the anteriority × response interaction. Results showed that ERPs corresponding to high ratings in both recent and lifetime exposure tasks were more positive than those of low ratings of both tasks on the centroposterior ROIs, t (46) = 1.96, p = 0.028, d = 0.29, one-tailed, but not on the anterior ROIs, t (46) = −1.19, p = 0.88, d = −0.17, one-tailed.

A quantitative comparison of the topographies with a range-normalization method^18^ revealed no statistical difference between the two tasks. For each participant, we first computed the difference ERPs between high and low responses within each task, then range-normalized these difference ERPs across all electrodes within each task. An ANOVA on range-normalized LPC voltage differences with factors anteriority, laterality, and task showed that the topographies were not significantly different between the two tasks: anteriority × task F (1, 46) = 0.58, p = 0.45, ${\hat{\eta }}_{G}^{2}$ = 0.002; laterality × task F (1, 46) = 1.85, p = 0.18,$\,{\hat{\eta }}_{G}^{2}$ = 0.003; anteriority × laterality × task F (1, 46) = 2.01, p = 0.16, ${\hat{\eta }}_{G}^{2}$ = 0.001.

In summary, ERPs associated with high ratings in both recent and lifetime exposure tasks were more positive than those associated with low ratings in the 500 ms to 800 ms time window on the centroposterior ROIs. The direction and the topography of these ERP effects were consistent with the LPC component previously described in the literature on old–new effects in the recognition-memory literature (see^1^ for a review).

### Does the LPC track only task-relevant memory signals?

First, we tested whether the LPC effect for cumulative recent exposure is present only when judgements of this dimension are required. If that was the case, it should not track the amount of recent exposure during the study phase during which this dimension was irrelevant for the judgement at hand (i.e., about animacy). For the critical comparison in the study phase, we binned the last presentation of each stimulus that was presented once or three times (low) versus seven or nine times (high). This process resulted in an average of 43 trials entering the ERPs for both binned levels. The corresponding trial rejection rates were 13% for the “low recent-exposure” condition and 14% for the “high recent-exposure” condition. When these data were analyzed using an ANOVA, we observed an effect in the 500–800 ms time window related to the amount of recent exposure, namely a significant anteriority × laterality × presentation frequency interaction; F (1, 47) = 6.82, p = 0.012, ${\hat{\eta }}_{G}^{2}$ < 0.001. Post hoc t-tests on the interaction revealed, however, that unlike the LPC effect observed in the test phase in posterior ROIs, the effect in this time window at study showed a right-lateralized frontal distribution: right anterior ROI, t (47) = 2.39, p = 0.02, d = 0.35, one-tailed; right centroposterior ROI, t (47) = 1.00, p = 0.16, d = 0.14.

**Figure 3:**
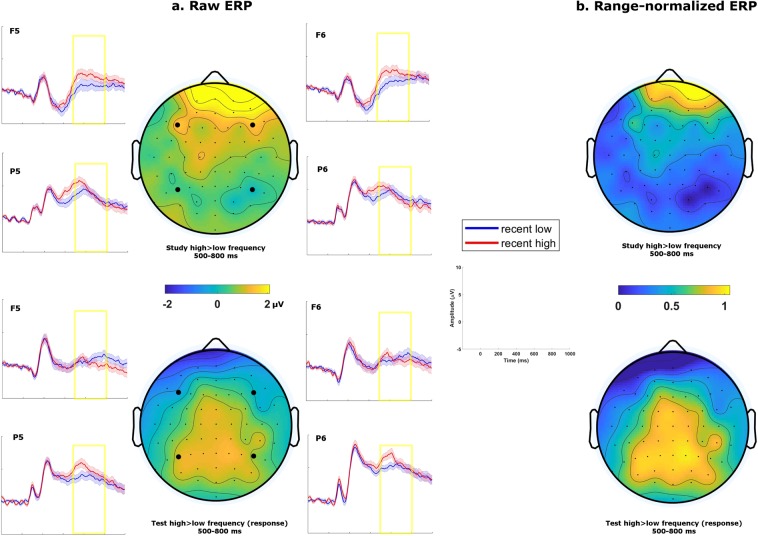
(**a**) Grand average topographies and ERP traces for recent exposures in the 500–800 ms time window. Traces are plotted for 4 representative electrodes, with shaded areas representing standard errors of the mean. Study phase contrasts (top) were generated with actual presentation frequency. Test phase (bottom) contrasts were generated with participants’ frequency responses. (**b**) Grand average topographies range-normalized across electrodes (i.e. values ranging from 0 to 1) of the same contrasts.

For a formal comparison of the topography of the effect in this time window between the two experimental phases (study versus test), we also conducted an ANOVA on the range-normalized difference ERP in the LPC time window with factors anteriority, laterality, and phase (study versus test). Critically, there was a significant interaction between anteriority and phase: F (1, 42) = 10.18, p = 0.003, ${\hat{\eta }}_{G}^{2}$ = 0.04, in line with the idea that the ERP component captured in this time window is not the same in both phases of the experiment (Fig. 3). In summary, the LPC we observed in relation to cumulative recent exposure was present only when such exposure was relevant to the current task.

In the next set of analyses, we tested whether the LPC effect we observed in relation to judgements of cumulative lifetime exposure was also present only when the task required consideration of this dimension. We compared the influence of cumulative lifetime exposure on ERPs during judgements of lifetime versus recent exposure. For the latter, we used normative estimates of lifetime exposure and the same binning with two levels (high versus low) as described previously. This process resulted in an average of 46 trials entering the ERPs for the “low lifetime-exposure” condition and 38 trials for the “high lifetime-exposure” condition. On average both conditions had 14% of the trials rejected. Critically, an ANOVA comparing the effect in the LPC time window between both tasks revealed a significant three-way interaction of anteriority × lifetime exposure × task, F (1, 46) = 4.76, p = 0.034, ${\hat{\eta }}_{G}^{2}$ = 0.001. Follow-up analyses of this three-way interaction in the LPC time window showed that a anteriority × lifetime exposure interaction during judgements of lifetime exposure, F (1, 46) = 15.37, p < 0.001, ${\hat{\eta }}_{G}^{2}$ = 0.008, where the differences in ERP amplitude between high versus low lifetime judgements were numerically more positive on the centroposterior ROIs, although the differences did not reach significance when tested using separate t-tests, all ps > 0.1. During judgements of recent exposure, no effect involving the factor “lifetime exposure” was significant, all ps > 0.1.

**Figure 4:**
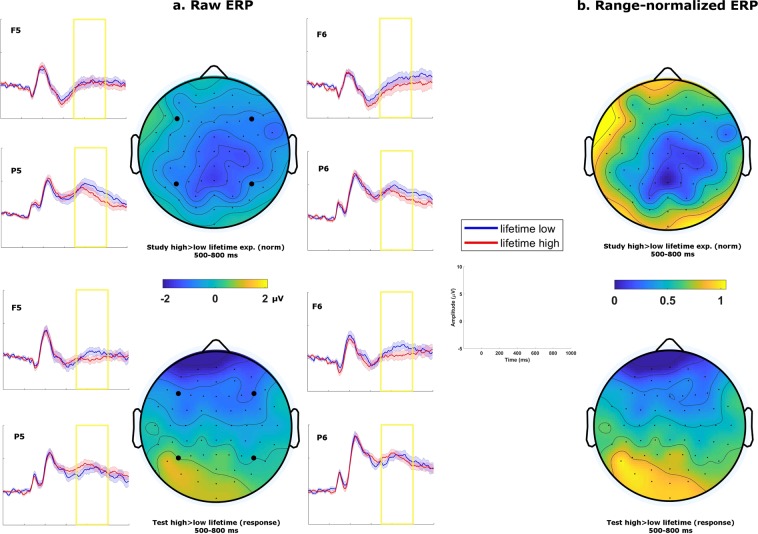
(**a**) Grand average topographies and ERP traces for lifetime exposures in the 500–800 ms time window. Traces are plotted for 4 representative electrodes, with shaded areas representing standard errors of the mean. Study phase contrasts (top) were generated with normative score. Test phase contrasts (bottom) were generated with participants’ responses on lifetime exposure. (**b**) Grand average topographies range-normalized across electrodes (i.e. values ranging from 0 to 1) of the same contrasts.

We also examined the issue of decision relevance in a similar comparison but focusing on the effect of lifetime exposure in the study phase (rather than presentation frequency; Fig. 4). The pattern of results was comparable to that of the previous analyses. When comparing the normative lifetime exposure ratings of first presentations in the study phase, we found a marginally significant main effect of lifetime exposure in the LPC time window, F (1, 42) = 3.94, p = 0.054, ${\hat{\eta }}_{G}^{2}$ = 0.007. Importantly, this effect differed topographically from the LPC effect observed in the test phase when participants made judgements on lifetime exposure, as indicated by a significant anteriority × laterality × phase interaction when tested using the range-normalization method described previously, F (1, 42) = 7.86, p = 0.008, ${\hat{\eta }}_{G}^{2}$ = 0.007. In summary, these analyses suggest that the LPC we observed in relation to lifetime exposure was present only when this dimension was relevant to the task.

### Could the LPC effects be explained based on differences in response times?

To examine whether the shifts in topography we report for the LPC, and interpret in relation to task relevance, could be explained by differences in RTs across trials or conditions, we conducted several additional sets of analyses. First, we added single-trial ERP analyses with a model comparison approach, separately for the recent and lifetime exposure effect, using the lme4 package in R^19^ (see^20^ for a similar approach). In these single-trial analyses, the mean amplitude in the 500–800 ms (LPC) time window was spatially averaged across electrodes in each ROI and was the dependent variable. The corresponding dataset includes all trials from the study and the test phase in which a response was made. The fixed factors were anteriority (frontal, centroparietal), laterality (left, right), task (animacy, frequency, lifetime), frequency (5-point), lifetime exposure (5-point), and RT (continuous). Random intercepts of participants and words were also modeled. Frequency of recent exposure was modeled as the actual presentation frequency, re-coded into 5 levels, in the study phase and in the lifetime exposure task; in the frequency task it was modeled as judged frequency on the 5-point scale employed. Lifetime exposure in the study phase and in the frequency judgement task was modeled based on normative data, while in the lifetime exposure task it reflects perceived degree of lifetime exposure judged on a 5-point scale (see Supplementary Materials for additional details on models). The resulting model comparisons were evaluated in terms of fits with χ^2^ tests among models. Critically, these analyses revealed that models with the factor frequency (Model 2) or lifetime exposure (Model 3) fit the data significantly better than a model that included only RT (Model 1), χ^2^(51) = 194.76, p < 0.001; and χ^2^(67) = 97.22, p < 0.001, respectively. These results suggest that the LPC effect we report cannot be explained solely by RT.

Second, we examined the role of RT in our topography ANOVA results. For each participant, we computed the average RT difference between high and low frequency trials, as well as the average RT difference between high and low lifetime exposure trials, separately for the study and the test phase. Subsequently, we used these RT differences as a covariate in the two relevant analyses that compared LPC topography of the frequency effect and lifetime exposure effect between the study and the test phase. Critically, the anteriority × phase (study or test) interaction remained statistically significant with inclusion of this covariate, F (1,42) = 10.18, p = 0.003; F (1,42) = 13.21, p < 0.001, for frequency and lifetime exposure, respectively. Again, these results suggest that the LPC effect we report cannot be explained solely with respect to differences in RT.

### Does source activity in the left lateral posterior parietal lobe follow the pattern of the LPC effect?

**Figure 5:**
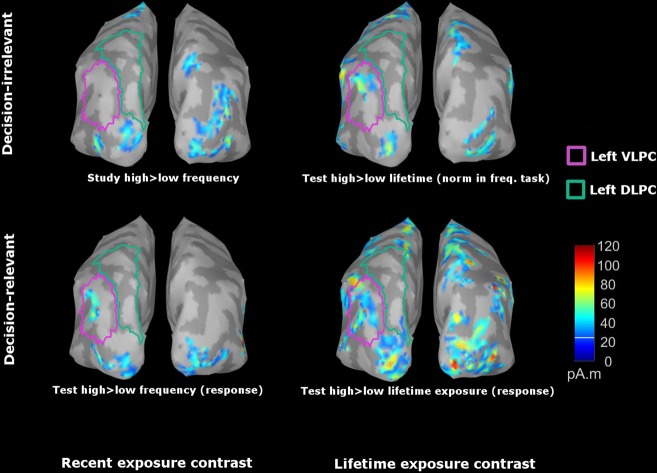
Grand average current density maps during the LPC time window for the contrasts of recent and lifetime exposure. Boundaries of left ventral and dorsal lateral parietal cortex are marked in purple and green, respectively. Maps were plotted with amplitude threshold of 20% and size threshold of 20 in Brainstorm.

**Figure 6:**
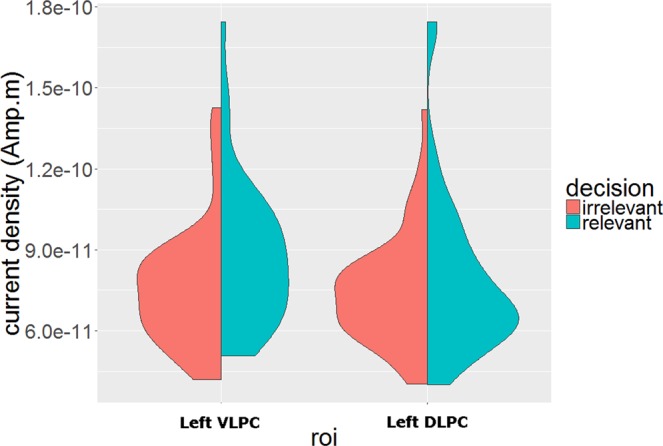
Violin plot on the effect of decision-relevance in the two ROIs (i.e. left ventral and dorsal lateral parietal cortices).

To examine cortical regions linked to the decision-dependent LPC effect, we performed source localization on the binned ERP data (i.e. high vs. low). A recent review of a large number of fMRI studies suggests a critical role of ventral lateral parietal cortex in making memory decisions^15^. Meanwhile, two electrophysiological studies also point to the potential contribution of surrounding areas, such as intra-parietal sulcus and superior parietal lobule^14,16^. These effects are typically left-lateralized. In light of these findings, we focused on two ROIs in the left lateral posterior parietal lobe, the ventral lateral parietal region which includes the angular gyrus, and the dorsal lateral parietal regions which includes the superior parietal lobule. The omnibus test was a repeated-measures ANOVA (n = 43). We extracted differences in absolute current densities between high and low bins, then averaged each difference across sources in each ROI and across the LPC time window (500 to 800 ms). These spatially and temporally averaged difference source measurements were used as the dependent variable, while decision relevance, task, and ROIs were used as within-participant independent variables. Significant main effects of decision-relevance and ROIs were observed, F (1, 42) = 6.88, p = 0.012, ${\hat{\eta }}_{G}^{2}=0.014$, F (1, 42) = 5.31, p = 0.026, ${\hat{\eta }}_{G}^{2}$ = 0.006, respectively. A two-way interaction between ROIs and decision-relevance was also observed, F (1, 42) = 9.48, p = 0.004, ${\hat{\eta }}_{G}^{2}$ = 0.004. We performed follow-up tests on this interaction, focusing on the effect of decision-relevance within each ROI. Larger differences in source activity for the decision-relevant compared to the decision-irrelevant contrast was observed in the left ventral lateral parietal ROI, t (42) = 3.58, p < 0.001, d = 0.55, but not in the left dorsal lateral parietal ROI, t (42) = 1.09, p = 0.28, d = 0.17 (Figs 5 and 6).

### Does the FN400 also track perceived prior exposure in a decision-relevant manner?

To determine whether the pattern of LPC effects was specific to this ERP component, we conducted a similar set of analyses to examine the time window of the FN400, that is, the other ERP component that has sometimes been reported to distinguish between perceived old and new items in recognition-memory judgements (although this remains controversial; see^3,4,6^). Starting with the omnibus ANOVA for recent- and lifetime-exposure judgements during the test phase, we found a trend similar to the effect in the LPC time window, as indicated by a two-way anteriority × response interaction, F (1,46) = 4.04, p = 0.050, ${\hat{\eta }}_{G}^{2}$ < 0.001. We also found a 4-way interaction of anteriority × laterality × task × response, F (1, 46) = 7.08, p = 0.011, ${\hat{\eta }}_{G}^{2}$ < 0.001. We further tested the 2-way interaction with simple-effect tests and the 4-way interaction with range-normalized topography comparison between the two tasks. Post-hoc tests on the anteriority × response interaction showed that the ERPs corresponding to the high response category were significantly more positive than those corresponding to the low response category on the centroposterior ROIs, t (46) = 2.22, p = 0.031, d = 0.32, one-tailed, but not on anterior ROIs, t (46) = 0.14, p = 0.44, d = 0.02. Topography comparison between the two tasks revealed a 3-way anteriority × laterality × task interaction, F (1, 46) = 5.73, p = 0.021, ${\hat{\eta }}_{G}^{2}$ = 0.003. While the direction of the effect in this time window is consistent with previously reported old-new effects^21,22^, we note that the scalp distribution in the lifetime exposure task was more posterior than a typical FN400 effect and more similar to an N400 as reported in studies on semantic memory^23^.

**Figure 7:**
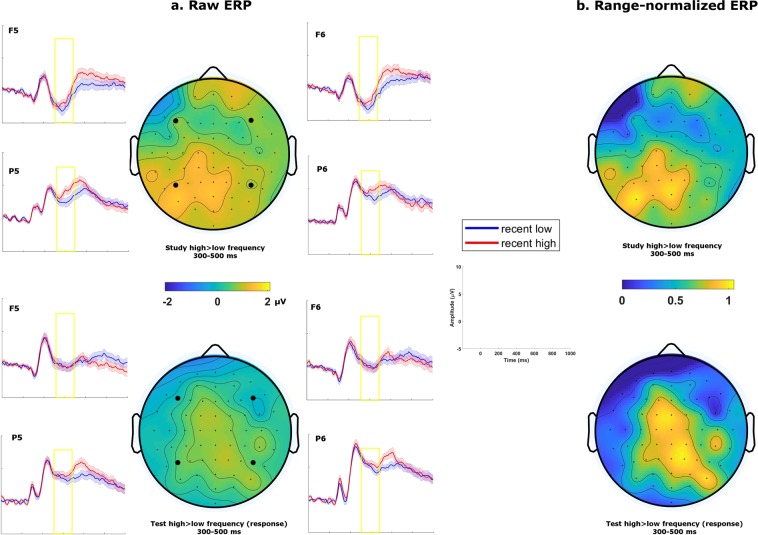
(**a**) Grand average topographies and ERP traces for recent exposures in the 300–500 ms time window. Traces are plotted for 4 representative electrodes, with shaded areas representing standard errors of the mean. Study phase contrasts (top) were generated with actual presentation frequency. Test phase (bottom) contrasts were generated with participants’ frequency responses. (**b**) Grand average topographies range-normalized across electrodes (i.e. values ranging from 0 to 1) of the same contrasts.

To determine whether the effect observed in the FN400/N400 time window (300–500 ms) for recent exposure was tied specifically to a condition in which this dimension is decision-relevant, we examined whether the effect was also present in the study phase. We observed a significant anteriority × presentation frequency interaction, F (1, 47) = 4.45, p = 0.040, ${\hat{\eta }}_{G}^{2}$ = 0.001; and a significant anteriority × laterality × presentation frequency interaction, F (1, 47) = 6.74, p = 0.013, ${\hat{\eta }}_{G}^{2}$ < 0.001. Post hoc t-tests on the 3-way interaction showed that the recent-exposure effect during the study phase was significant on the left centroposterior ROI, t (47) = 2.67, p = 0.010, d = 0.38, but not in the anterior ROI, t (47) = 1.04, p = 0.15, d = 0.15. A topography comparison of the recent-exposure effect between the study and the test phase revealed a significant three-way interaction, anteriority × laterality × phase, F (1, 42) = 8.23, p = 0.006, ${\hat{\eta }}_{G}^{2}$ = 0.005. The effect of cumulative recent-exposure in this time window appeared to be more pronounced on the left centroposterior ROI during the study phase (Fig. 7).

**Figure 8:**
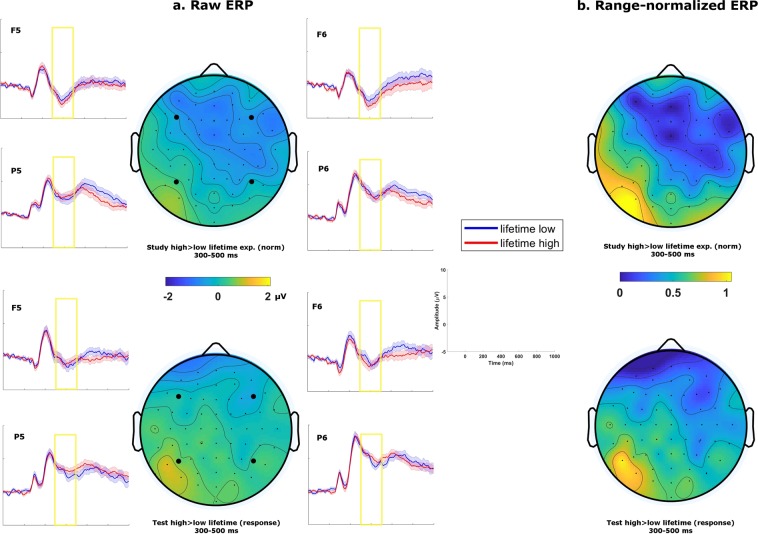
(**a**) Grand average topographies and ERP traces for lifetime exposures in the 300–500 ms time window. Traces are plotted for 4 representative electrodes, with shaded areas representing standard errors of the mean. Study phase contrasts (top) were generated with normative score. Test phase contrasts (bottom) were generated with participants’ responses on lifetime exposure. (**b**) Grand average topographies range-normalized across electrodes (i.e. values ranging from 0 to 1) of the same contrasts.

In our final analysis, we addressed whether effects of lifetime exposure on the FN400/N400 are decision relevant, first by comparing ERPs for judgements of lifetime exposure with ERPs for normative lifetime exposure during frequency judgements of recent exposure. Critically, unlike for the LPC, there was no significant interactions involving lifetime exposure × task in the 300–500 ms time window, all ps > 0.08. We also examined decision relevance in a similar comparison but focusing on the effect of lifetime exposure in the study phase (rather than presentation frequency). The pattern of results was comparable to that of the previous analyses. When comparing the ERPs elicited by first presentations of stimuli in the study phase, we found a significant three-way anteriority × laterality × normative lifetime exposure interaction, F (1, 42) = 6.61, p = 0.014, ${\hat{\eta }}_{G}^{2}$ < 0.001. However, a topography comparison showed that this effect did not differ significantly from the ERPs for judgements of lifetime exposure in the test phase (Fig. 8), anteriority × phase, F (1, 42) = 0.14, p = 0.71, ${\hat{\eta }}_{G}^{2}$ < 0.001, laterality × phase, F (1, 42) = 1.07, p = 0.31, ${\hat{\eta }}_{G}^{2}$ = 0.002, and anteriority × laterality × phase, F (1, 42) = 0.31, p = 0.58, ${\hat{\eta }}_{G}^{2}$ < 0.001.

### Peak-based analyses

We performed exploratory peak-based analyses on effects reported above. In sum, the peak-based results, based on local peak amplitudes as well as two-step PCA, are largely consistent with what we reported (see Supplementary Materials for more details).

## Discussion

We investigated whether the LPC is linked to memory judgements and tracks the strength of multiple types of memory signals in a flexible decision-dependent manner. Participants made memory judgements either on relative frequency of item exposure in a study phase or based on cumulative lifetime experience. We showed that the LPC tracks cumulative exposure regardless of whether the accumulation happened recently in the laboratory or over the lifetime. Critically, this effect is decision-dependent. It was present only when the memory judgement at hand required consideration of the relevant dimension. Moreover, source localization analyses revealed decision-dependent activity in left ventral lateral parietal cortex. Finally, we observed two topographically distinct components in an earlier time window that showed differential sensitivity to decision-relevance. The FN400 produced a pattern of activity similar to the LPC for recent cumulative exposure but not for lifetime exposure. Namely, it tracked recent cumulative exposure only when it was relevant to the decision. A related ERP component, the N400, tracked both recent and lifetime cumulative exposure, but it did so regardless of decision-relevance.

### Judgements of recent exposure to object concepts

Most prior research on the LPC has focused on some form of memory judgement about recent laboratory exposure in study-test paradigms. This research has shown that the LPC robustly distinguishes hits from correct rejections in recognition memory judgements^1^. A significant body of research suggests that the LPC is linked specifically to the contribution of episodic recollection to recognition memory judgements. Evidence supporting this interpretation comes from studies adopting a variety of paradigms designed to (more or less) selectively manipulate recollective processes, including source memory judgements^24^, associative recognition memory^25,26^, and the Remember/Know paradigm^2,27^. Specifically, the LPC has been shown to increase in size for correct versus incorrect source recognition judgements, for Remember as compared to Know responses, and for intact versus rearranged pairs in associative recognition. Some findings, however, have questioned the specific link between the LPC and recollection, and instead point to a broader role of the LPC that is tied to decision making in memory judgements.

Finnigan, Humphreys, Dennis, and Geffen^28^ analyzed the LPC amplitude in relation to the accuracy of old/new decisions in a recognition-memory paradigm, contrasting with the more common practice of focusing only on correct responses (i.e. Hit vs. Correct Rejection). They showed that the LPC was significantly more positive for correct than incorrect recognition responses not only when old but also when new items were considered. Moreover, correct rejections elicited a more positive LPC than did false alarms, indicating that this decision effect is not solely driven by general differences between old and new items (i.e., the old/new effect). More recently, as reviewed in the Introduction, a study based on drift-diffusion modeling of behavioural data revealed that the EEG amplitude in the time window coinciding with the LPC predicts participants’ reaction time and accuracy of recognition-memory decisions on a trial-by-trial basis^6^. Based on this finding, the authors suggested that the LPC tracks evidence accumulation in memory decisions. In other recent research directly focusing on familiarity and recollection, it has been reported that the LPC tracks confidence ratings when controlling for the relative proportion of Remember and Know responses^5^. Moreover, high-confidence Know responses elicited a more positive LPC than low-confidence Remember responses, a finding that the authors interpreted with reference to memory strength. Although no formal modeling of decision making was involved, this notion of memory strength can also be thought of as the evidence that drives memory decision. Findings from the current study are consistent with the idea that the LPC tracks memory signals when they are decision-relevant. Critically, they also suggest that it is not sensitive to just one type of memory signal, but instead it can track multiple signals in a flexible manner depending on the specific demands of the memory task at hand.

In the present study, we employed frequency judgements to show that the LPC tracks memory signals in judgements about recent laboratory exposure in a decision relevant manner. We manipulated the decision-relevance of frequency information by comparing the effect of frequency in the test and study phases. When comparing stimuli judged to have been presented with high versus low frequency, we observed the classic LPC during test. By contrast, when comparing the final presentation of stimuli presented 7 or 9 times in total versus once or 3 times in total, we did not observe an LPC in the study phase. In the earlier time window (i.e. 300–500 ms), a contrast based on judged presentation frequency in the test phase elicited a different effect compared to the contrast of actual presentation frequency in the study phase. This effect is comparable to the FN400 effect reported for old-new effects in other recognition paradigms that has been linked to relative familiarity^21^. As such our findings suggest that not all ERP components that have previously been linked to memory judgements are equally tied to decision processes.

Evidence from computational modeling and behavioral research on retrieval dynamics suggests that under many experimental conditions, familiarity is the primary basis for accurate frequency judgements^29^. The FN400 effect on frequency judgements observed in the current study is consistent with this view. We manipulated item exposure at study in small increments over a substantial range of presentations and, at test, we included no novel lures. With this design, we minimized the likelihood that contextual information (underlying recollection) would allow for differentiation of the frequency of recent exposures. Thus, we maximized the need to rely on graded signals that code for recent incremental changes in familiarity in our frequency judgements. Nevertheless, it has been proposed that recollection may also contribute to frequency judgements through recursive reminding^30^. Hintzman (2004) has argued for an account in which the repeated presentation of an item reminds participants of prior conscious experiences with that item. Consequently, both the current presentation and the recollected experiences are encoded, allowing for a recursive process. Frequency judgements are proposed to be sensitive to the depth of reminding, which naturally tracks the amount of cumulative recent exposure. Critically, the recursive reminding mechanism would operate not only when frequency is decision relevant in the test phase, but it would also occur spontaneously at study. The fact that we observed the LPC during frequency judgements in the test phase but not at study suggests that it does not reflect recollection-related recursive reminding. Findings from patient-based research also argue against an interpretation of the LPC in the current task as being uniquely tied to recollection. We have previously reported that a focal anterior temporal-lobe lesion that includes left perirhinal cortex, but spares the hippocampus, produced impairments in making frequency judgements on this task in an individual (NB) with documented familiarity impairments but preserved recollection abilities^13^.

### Judgements of lifetime exposure to object concepts

To our knowledge, the current study is the first to directly probe the LPC in memory judgements about cumulative lifetime exposure to concepts (but see^21^ for related research on effects of word frequency). Mirroring our results for cumulative recent exposure, we observed an LPC effect when comparing stimuli judged to have high versus low lifetime exposure and the dimension was, thus, decision-relevant. In contrast, when comparing stimuli with high versus low normative lifetime exposure during recent exposure or animacy judgements (in the study phase), the LPC effect was absent. Thus, these findings provide further support for the notion that the LPC tracks memory signals only to the extent that they are decision relevant.

A variable related to cumulative lifetime exposure of concepts that has also been examined in the psycholinguistics- and recognition-memory literature is word frequency, that is, the frequency of words as measured in linguistic corpora. High frequency words tend to elicit faster and more accurate responses in linguistic paradigms^31^. In recognition paradigms, however, high frequency words tend to elicit more incorrect responses (i.e. more misses and false alarms as often described as mirror effect)^21^. This behavioral effect, in particular the increase in false alarms for words with high frequency, has been suggested to reflect a reliance on absolute memory strength (or cumulative lifetime exposure) when no recent change in strength was introduced in a study phase^9,32^.

ERPs in both the FN400/N400 and the LPC time windows have been shown to be sensitive to word frequency across a range of tasks. Several studies have shown that high frequency words elicit a smaller N400^21,31,33,34^. The literature on the LPC effect of word frequency is more complex, in part due to inconsistencies in terminology (sometimes also referred to as P300^33^, P530^31^, or P600^34,35^) and choice of time window. Rugg^31^, for example, used words of different frequencies with a concurrent manipulation of experimental repetition in a lexical decision task. He reported a word frequency effect on the LPC that interacted with repetition, in that the LPC was more positive for high frequency words during the first presentation, but switched polarity and was more positive for low frequency words during the second presentation. Differing somewhat from those results, we observed that the ERPs in the LPC time window were more positive for words with low degree of lifetime exposure for the first presentation during the study phase. However, it is difficult to tell whether this subtle difference could be attributed to the selection of electrodes (Rugg reported a reversed polarity on the electrode Pz during the first presentation), differences between how word frequency and lifetime familiarity are represented in the brain, or differences between tasks (lexical decision vs animacy judgements).

In the context of our conclusion that the LPC marks decision-dependent memory processes, it also is interesting to ask whether word frequency would affect the LPC amplitude in memory tasks that require no judgement of word frequency or lifetime exposure. Extant results in the literature are mixed. On one hand, some studies have shown that word frequency interacts with study status (old/new) in modulating the LPC amplitude in recognition paradigms^35,36^. On the other hand, a recent study^21^ that focused on old/new recognition judgements reported no effect on the LPC when comparing correct rejections of words with high versus low frequency. Similarly, when we compared stimuli with high and low degree of lifetime exposure during the frequency judgement in the test phase, we observed no effect in the LPC time window. Although some caution is warranted when interpreting this negative finding, we note that it is in line with the idea that the LPC tracks memory signals only when they are decision relevant. On a more general level, it is also worth keeping in mind that experience with words and lifetime exposure to concepts are correlated but only moderately so^10^. In the lifetime exposure task, we specifically instructed participants to make their judgement based on the concepts a word refers to rather than word itself. While increases in lifetime exposure of concepts tend to be tied to variability in episodic context, and typically go hand in hand with increases in concept knowledge^7^, it is unclear whether the same holds for increases in exposure to words in a more restricted reading context.

Although the structure of the task we employed to probe lifetime familiarity did not require any reference to a specific episodic encounter, it is interesting to consider whether episodic recollection may still have impacted performance. This possibility deserves consideration in light of prior evidence that implicates episodic recollection and hippocampal functioning in ostensibly semantic tasks, such as object naming or conceptual fluency, that is, the speeded generation of exemplars from different semantic categories^37–42^. Such evidence has led to the suggestion that episodic and semantic memory may interact even on tasks that do not require recollection, and that recollection of a pertinent autobiographical episode can help generate or retrieve semantic information (see^40^ for detailed discussion). Behaviorally, ratings of degree of lifetime exposure, as used in the current study, are positively correlated with perceived ease of recovering a pertinent unique autobiographical episode^13^. However, we previously reported that a patient (HC) with severe hippocampal damage and documented impairments in recollection^43,44^ performed similarly to healthy controls on the same paradigm^13^. Furthermore, patient NB, an individual with well documented deficits in assessment of familiarity based on recent exposure, but preserved recollection abilities, showed abnormal performance in judging lifetime exposure to concepts^13^. This pattern of results suggests a functional distinction between the recollection of the time and place of particular autobiographical instances of object encounters, and the assessment of degrees of experience over hundreds or thousands of encounters throughout a lifetime. Critically, it also suggests that contributions of recollection to assessing cumulative lifetime exposure are neither necessary nor sufficient.

### Source localization of ERP effects and the role of the parietal lobe

The electrodes that were selected to represent the LPC in the current study are broadly sensitive to source activity in the posterior lateral parietal cortex and surrounding regions^45^. Recent studies have linked left posterior lateral parietal cortex to recognition memory decisions with intracranial electrocorticography (ECoG) and recordings from depth electrodes in humans^14–16^. To explore whether the decision-dependent LPC effect could be linked to activity in this region, we employed source localization to estimate the current densities in the left ventral and dorsal posterior lateral parietal lobe. Because we observed the decision-dependent effect on the LPC in both types of cumulative memory judgements, we examined the data for both tasks in the same analysis. When contrasting high versus low exposure stimuli across tasks, source activation in the left ventral lateral parietal cortex was indeed stronger for the decision-relevant contrast than the decision-irrelevant contrast during the LPC time window. This effect was not present in dorsal parietal cortex. While caution is necessary when interpreting our source localization results in relation to specific anatomical structures, we note that the findings from numerous fMRI studies point to a role of the left angular gyrus in aspects of decision making during memory judgement (see^15^ for a review), and that the interpretation of this angular gyrus involvement mirrors that of the LPC in ERP studies. To the extent that source activity in the left angular gyrus during the LPC time window was found to be sensitive to the decision-relevance of information in the current study, our findings provide initial evidence that links these sets of findings across the two imaging methodologies. Future research can build on these initial source-localization findings with an approach that combines both imaging modalities with the tasks employed in the present study.

## Method

### Participants

Sixty-five participants (38 females) were recruited through posters or an online recruitment tool^46^. All participants were 18 to 35 years old, right handed, native English speakers who had lived in Canada since childhood. None of them reported any known psychiatric or neurological disorder. Seven participants were excluded from final analyses due to technical problems with EEG equipment. One additional participant was excluded because they failed to follow instructions. Other analysis-specific exclusions were applied, such that participants with less than 10 trials in any of the experimental conditions were not considered in corresponding ERP analyses. Depending on the phases, the exclusion criteria removed 9 or 10 of 57 participants, resulted in 47 participants for analyses of the test phase, and 48 participants for the study phase. However, for analyses including the within-participant experiment phase (i.e. study or test) factor, only 43 participants whose data from both phases passed the criteria were included.

The study was approved by the Western University Non-Medical Research Ethics Board (NMREB). Informed consent was acquired from each participant before the experiment. Participants were given course credit or monetary compensation. All experiments were performed in accordance with the approved guidelines and regulations.

### Material

Stimuli were 250 concrete English nouns selected from a normative database collected from Canadian participants^17^. They were divided into 10 bins of 25 words (Table 3). Five bins were randomly selected to be used in the study phase and the cumulative recent-exposure task (i.e. relative frequency-judgement), and the other 5 bins were used in the lifetime exposure task (see Procedure). This assignment was counterbalanced across participants to create two versions of the experiment. Word length, number of phonemes, number of syllables, word frequency, and normative lifetime exposure ratings were matched across bins as verified by an ANOVA. Stimuli were selected to cover a wide range of lifetime exposure ratings in the database. On the 9-point scale of lifetime exposure provided by the database, the two versions of the experiment had mean ratings of 5.44 and 5.52, and ranges of 7.00 and 6.90.

**Table 3:** Average normative lifetime exposure, concreteness, natural log of word frequency, number of letters, and number of phonemes for the ten bins selected as stimuli.

	Bin1	Bin2	Bin3	Bin4	Bin5	Bin6	Bin7	Bin8	Bin9	Bin10
Normative lifetime exposure	5.20	5.85	5.64	5.01	5.49	5.19	5.44	5.57	5.61	5.81
concreteness	4.84	4.83	4.73	4.82	4.88	4.80	4.87	4.85	4.87	4.84
ln(KF)	1.85	1.76	1.63	1.79	1.48	1.35	1.65	1.62	1.66	1.66
Number of letters	6.12	5.92	6.28	5.68	6.00	6.00	5.84	6.36	5.88	5.60
Number of phonemes	4.64	5.00	5.24	4.72	4.96	5.00	4.80	5.08	4.80	4.92

Concreteness ratings were taken from Brysbaert *et al*.^59^. Other measurements were taken from McRae, Cree, Seidenberg, & McNorgan (2005).

### Procedure

**Figure 9:**
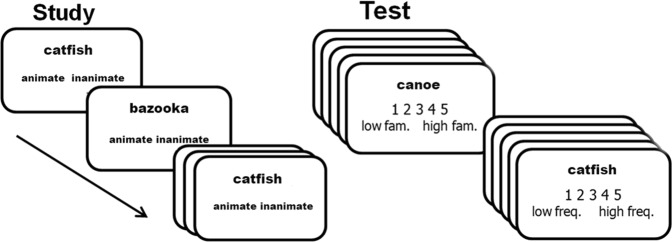
Experimental procedure. Note that the text below the scale in the test phase is only for illustrative purposes, they were not shown to participants in the actual experiment.

After acquiring informed consent, participants were seated in front of a monitor in a soundproof booth. Oral instructions were given to participants regarding the general structure of the study phase. Participants were instructed to minimize movements and remain vigilant throughout the experiment. Written instructions about response-key mappings were displayed on the monitor for participants to read at their own pace. E-prime^47^ was used to present the stimuli and log behavioral responses. For the study phase, a list of 125 unique concrete nouns (i.e. 5 bins) appeared on the monitor one at a time following a fixation cross. The stimuli were presented at different frequencies (i.e., number of repetitions) across bins, such that items were presented either one (bin 1), three (bin 2), five (bin 3), seven (bin 4), or nine times (bin 5). In sum, this resulted in 625 randomized trials in the study phase. For each trial, a fixation cross was presented for 1000 milliseconds, and became bolded for 1000 milliseconds to indicate the imminent presentation of a stimulus. A stimulus and the response options then were presented for 1000 milliseconds, and participants were asked to judge the animacy of each word by pressing one of two keys (Fig. 9). They were not told about the ensuing memory test phase.

Immediately after the study phase, participants were given oral instructions about the structure of the test phase. Written instructions regarding response key-mappings were displayed on the monitor for participants to read at their own pace. The test phase consisted of two types of trials: recent and lifetime exposure judgements. For the recent exposure task, participants judged the relative presentation frequency of each word in the study phase on a 5-point scale. For the lifetime exposure task, participants were presented with unstudied stimuli (i.e. the other 5 bins) and were asked to judge how familiar each corresponding concept was based on their lifetime experience, on a 5-point scale. There were 125 trials per task. The two tasks alternated in blocks every 5 trials. A message indicating the task type for the next 5 trials was shown for 2000 milliseconds prior to every alternation. The presentation order of items was randomized for each task. For both tasks, each trial started with a fixation cross, which was presented for 1000 milliseconds and was subsequently bolded for 1000 milliseconds to indicate the imminent presentation of a stimulus. Then a stimulus and the response options were presented for 2500 milliseconds during which participants’ responses were registered. Participants were asked to use all 5 keys and both hands. The mapping of the keys was counterbalanced across participants such that for approximately half of the participants, “5” was mapped on the left of the monitor and the keyboard, while for the other half, “5” was mapped on the right. Each key was mapped onto one finger. From left to right, participants used their left middle finger, left index finger, right index finger, right middle finger, and right ring finger to press corresponding keys.

### Behavioral data collection and preprocessing

To quantify participants’ behavioral performance, their ratings in the recent and lifetime exposure tasks were correlated with the actual presentation frequencies in the study phase and the normative lifetime exposure ratings from the database by McRae *et al*. (2005), respectively. On trials in which a participant failed to provide a response, the participant’s averaged response for the corresponding judgement type (i.e. recent/lifetime exposure) was used.

### ERP data collection and preprocessing

EEG data were collected using a Biosemi ActiveTwo 64-channel system. Electrode placements followed the international 10–20 system (“Biosemi Headcaps”)^48^. Two extra electrodes were applied on bilateral mastoids to be used in offline re-referencing. Another four extra electrodes were applied to the lateral corners of both eyes, above and below the left eye, to capture eye movements. Electrode offsets were kept below 20 mV. The data were originally sampled at 2048 Hz, and were down-sampled to 512 Hz to be imported into EEGLAB^49^, a free toolbox for MATLAB (MATLAB-R2015a, The MathWorks, Inc.)^50^. Data for malfunctioning electrodes were interpolated from neighboring electrodes using the spherical interpolation algorithm provided in EEGLab. For study phase data, four participants had one electrode interpolated, and one participant had two electrodes interpolated. For the test phase data, five participants had one electrode interpolated, and one participant had two electrodes interpolated. All other participants had no interpolated electrodes. Data were bandpass filtered between 0.1 to 30 Hz. An independent component analysis (ICA) was applied to identify and remove ocular artifacts^51^. The data were then re-referenced to linked mastoids. Epochs were extracted from −199 ms to 998 ms with reference to stimulus onsets. A moving window with a width of 200 ms, a step size of 100 ms, and a threshold of 100 μV was used to mark remaining artifacts in the epoched data. Data were then averaged with respect to trial types (i.e., experimental task and response selected) to extract ERPs. All marked epochs were excluded from the averaging process. After artifact rejection, participants with less than 10 trials in any condition were excluded from statistical analyses. On average 43 trials contributed to each ERP, which corresponds to a rejection rate of 13%.

To probe for any experimental effects in the ERP recordings, four regions of interest (ROI) were selected. These ROIs were Left Anterior (Fp1, AF3, AF7, F1, F3, F5, F7, FC1, FC3, FC5, FT7), Right Anterior (Fp2, AF4, AF8, F2, F4, F6, F8, FC2, FC4, FC6, FT8), Left Centroposterior (C1, C3, C5, T7, CP1, CP3, CP5, TP7, P1, P3, P5, P7, P9, PO3, PO7, O1), and Right Centroposterior (C2, C4, C6, T8, CP2, CP4, CP6, TP8, P2, P4, P6, P8, P10, PO4, PO8, O2). ERP data were averaged across electrodes within each ROI before being submitted to statistical analyses. Following extant research (e.g.^2,22^), the LPC and FN400/N400 time windows were chosen a priori to be 500–800 ms and 300–500 ms, respectively. Omnibus ANOVAs were carried out with the mean amplitude within each time window as the dependent variable, violations of the sphericity assumption were corrected by the Greenhouse-Geisser procedure. Multiple comparisons were corrected using the Bonferroni procedure in all tests following the omnibus ANOVAs. All p-values are reported following these corrections, unless otherwise specified. Effect sizes are reported using generalized eta squared: ${\hat{\eta }}_{G}^{2}$^52^ and Cohen’s d for F-tests and t-tests, respectively.

### LPC source localization

The exploratory source localization was carried out using the Brainstorm MATLAB toolbox^53^. We used default anatomy that is based on Colin 27 atlas^54^ for all participants. The electrode locations were imported from BioSemi 64 10–10 cap file provided by the Brainstorm. The forward model was estimated using OpenMEEG BEM^55,56^. The inverse solution was estimated using Tikhonov-regularized minimum-norm^57^, with current density as the measurement. The estimated dipole orientations were constrained to be normal to the cortex. For each participant, noise and data covariance matrices used in the inverse solution were computed from the epoched EEG data separately for the study and the test phase, using pre-stimulus baseline (−199.20 ms to −2.00 ms) and roughly the first second of stimulus presentation (0.00 ms to 998.00 ms). The source localization was performed on the binned ERPs in each condition (high vs. low, see Results), and the difference in absolute current densities between the high and low bins were extracted for inferential statistics. We focused on the left dorsal and ventral lateral posterior parietal lobe, which were defined using the Desikan-Killiany atlas^58^ offered in the Brainstorm Scout module.

## Supplementary information

Supplementary Materials

## Data Availability

The datasets generated during and/or analysed during the current study are available from the corresponding author on reasonable request.
