# Supplementary material for: Late positive complex in event-related potentials tracks memory signals when they are decision relevant
Source: Sci Rep. 2019 Jul 1;9:9469. doi: 10.1038/s41598-019-45880-y (PMC6603184; doi:10.1038/s41598-019-45880-y)
Supplement: Supplementary file 1 — Supplementary Materials [file 41598_2019_45880_MOESM1_ESM.docx]

**Late positive complex in event-related potentials tracks memory signals when they are decision relevant**

**Haopei Yang^1,*^, Geoffrey Laforge^2^ , Bobby Stojanoski^2^ , Emily S. Nichols^3^ , Ken McRae^2^ , and Stefan Köhler^2,4,+^**

^1^Brain and Mind Institute, Department of Neuroscience, London, N6A 3K7, Canada

^2^Brain and Mind Institute, Department of Psychology, London, N6A 3K7, Canada

^3^Brain and Mind Institute, Department of Physics, London, N6A 3K7, Canada

^4^Rotman Research Institute, Baycrest Centre, Toronto, M6A 2X8, Canada

[*haozi078@gmail.com](mailto:*haozi078@gmail.com)

+stefank@uwo.ca

# **R code for single-trial modeling with reaction time**

#LPC

library(rio)

myLPC=import("C:\\Users\\haozi\\Desktop\\paper_1\\revision_1\\single_trial\\43Ss_singletrial_studytest_LPC.csv")

myLPC$ant_pos=as.factor(myLPC$ant_pos)#ROI anteriority 2-level

myLPC$left_right=as.factor(myLPC$left_right)#ROI laterality 2-level

myLPC$SSID=as.factor(myLPC$SSID)#participant ID

myLPC$freq=as.factor(myLPC$freq)#recent exposure 5-level

myLPC$fam=as.factor(myLPC$fam)#lifetime exposure 5-level

myLPC$task=as.factor(myLPC$task)#animacy judgement (study), frequency, or lifetime

myLPC$animacy=as.factor(myLPC$animacy)#whether a concept is animate 2-level

myLPC$word=as.factor(myLPC$word)#unique word ID

newLPC=subset(myLPC,RT>0)#delete trials with no resp

#compare model of only RT and model with RT and effects of interest(freq,fam,etc.)

library(lme4)

RTmd=lmer(value~ant_pos*left_right*RT+(1|SSID)+(1|word),data=newLPC)

RTandFREQ=lmer(value~ant_pos*left_right*RT+ant_pos*left_right*freq*task+(1|SSID)+(1|word),data=newLPC)

anova(RTmd,RTandFREQ)

RTandFAM=lmer(value~ant_pos*left_right*RT+ant_pos*left_right*fam*task+(1|SSID)+(1|word),data=newLPC)

anova(RTmd,RTandFAM)

# **Local peak amplitudes and two-step PCA**

We first measured local peak amplitude of the ERPs using ERPLAB within the two time windows typically examined for the LPC and FN400/N400. Specifically, the LPC peak was sought as a positive local peak within the window of 500 to 800 ms. And the FN400/N400 peak was sought as a negative local peak within the window of 300 to 500 ms.

Secondly, we also performed two-step PCA (temporal then spatial) on the ERP data following the guidelines in Dien (2012) using the ERP PCA toolkit (Dien, 2010). The data were down-sampled to 128 Hz due to RAM limitations. The number of factors retained was determined through a parallel test. Factors representing the two ERP of interest were chosen through visual inspection guided by previous literature. The LPC was defined as a positive peak with a parietally-centered topography within the time window of 400 to 800 ms. The FN400/N400 was defined as a negative peak with a (frontal) parietal distribution within the time window of 300 to 500 ms. The LPC time window was relaxed to allow overlapping with the FN400/N400 time window in PCA analyses as the procedure is designed to separate temporally overlapping components. Factors were then fed to the autoPCA process to extract peak channel and peak value. These values for selected factors that represent either LPC or FN400/N400 served as inputs to a robust ANOVA for inferential statistics.

Below we present a table showing the outcomes of these analyses in comparison with our preferred, previously reported methods (mean amplitude with a priori selected time window, local peak amplitude with a priori selected time window, and PCA), along with a figure illustrating the Principal Components linked to the ERP effect we reported in the manuscript. The same ANOVA models were used across mean amplitude and local peak measurements. For the PCA-based analyses, we also report the peak latency and the peak electrode as produced by the autoPCA process. To aid this comparison, we present them in the order as they appear(-ed) in the manuscript with corresponding current page numbers. Please note that there a few empty cells for PCA based analyses as they could not be computed and would be largely redundant after the spatial PCA step.

*Table S1. Statistical results across three different measurement types on key contrasts.*

|  | Mean amplitude (as reported in previous versions) | Local peak | PCA |
| --- | --- | --- | --- |
| 1. ANOVA on the LPC amplitude in the test phase (p. 6) | anteriority x response: F (1, 46) = 18.96, p < .001, $\hat{\eta}_{G}^{2}$ = .005 | anteriority x response: F (1, 46) = 21.86, p < .001, $\hat{\eta}_{G}^{2}$ = .007 | 531 ms to 539 ms, on electrode PO3, main effect of response, T_WJt_/c (1.0, 42.0) = 20.59, p < .0001 (figure S1a) |
| 1. Post-hoc t-tests on 1. Response effect in each level of anteriority (p. 6) | Centroposterior: t (46) = 1.96, p = .028, d = 0.29  Anterior: t (46) = -1.19, p = .88, d = -0.17 | Centroposterior: t (46) = 1.40, p = .085, d = 0.20  Anterior: t (46) = -1.72, p = .95, d = -0.25 |  |
| 1. LPC topographical comparison between the two tasks in the test phase (p. 7) | No effect involving the task factor returned significance, all ps > .15 | No effect involving the task factor returned significance, all ps > .20 |  |
| 1. Study phase frequency effect in the LPC time window (p . 7) | anteriority × laterality × presentation frequency interaction; F (1, 47) = 6.82, p = .012, $\hat{\eta}_{G}^{2}$ < .001 | anteriority × laterality × presentation frequency interaction; F (1, 47) = 1.65, p = .20, $\hat{\eta}_{G}^{2}$ < .001  However, there is a marginally significant main effect of frequency, F (1, 47) = 3.98, p = .052, $\hat{\eta}_{G}^{2}$ = .017 | 594 ms to 602 ms, on electrode Cz, significant presentation frequency effect, T_WJt_/c (1.0,43.0) = 4.96, p = .031.(figure S1b) |
| 1. Topographical comparison between the study and the test phase frequency effect in the LPC window (p. 7) | anteriority × phase: F (1, 42) = 10.18, p = .003, $\hat{\eta}_{G}^{2}$ = .04 | anteriority × phase: F (1, 42) = 7.76, p = .008, $\hat{\eta}_{G}^{2}$ = .024 |  |
| 1. Comparing LPC lifetime exposure effect between the two tasks in the test phase (p. 8) | anteriority × lifetime exposure × task: F (1, 46) = 4.76, p = .034, $\hat{\eta}_{G}^{2}$ = .001 | anteriority × lifetime exposure × task: F (1, 46) = 4.98, p = .031, $\hat{\eta}_{G}^{2}$ = .002 | 484 ms to 492ms, on electrode PO3, significant task × lifetime exposure: T_WJt_/c (1.0,42.0) = 5.12, p = .027. (figure S1c) |
| 1. Post-hoc ANOVA on 6. LPC lifetime exposure effect in each task (p. 8) | During lifetime exposure judgement: anteriority × lifetime exposure: F (1, 46) = 15.37, p < .001, $\hat{\eta}_{G}^{2}$ = .008  During recent exposure judgement: no effect involving the factor “lifetime exposure” was significant, all ps > .1 | During lifetime exposure judgement: anteriority × lifetime exposure: F (1, 46) = 10.90, p = .002, $\hat{\eta}_{G}^{2}$ = .008  During recent exposure judgement: no effect involving the factor “lifetime exposure” was significant, all ps > .1 | During lifetime exposure judgement, significant lifetime exposure effect: T_WJt_/c (1.0,42.0) = 17.11, p < 0.001. (figure S1c)  During recent exposure judgement, nonsignificant lifetime exposure effect: T_WJt_/c (1.0,42.0) = 0.40, p = .53. (figure S1c) |
| 1. Lifetime exposure effect in the study phase in the LPC time window (p. 8) | marginally significant main effect of lifetime exposure in the LPC time window, F (1, 42) = 3.94, p = .054, $\hat{\eta}_{G}^{2}$ = .007 | Significant main effect of lifetime exposure in the LPC time window, F (1, 42) = 7.00, p = .011, $\hat{\eta}_{G}^{2}$ = .014 | 484 ms to 492 ms, on electrode Cz, significant lifetime exposure effect, T_WJt_/c (1.0,43.0) = 8.30, p = .0054. (figure S1d)  656 ms to 664 ms, on electrode POz, significant lifetime exposure effect, T_WJt_/c (1.0,43.0) = 8.01, p = .0063. (figure S1e)  Note that in both principle components, stimuli with low degree of lifetime exposure evoked more positive amplitude, which is consistent with the ERP results. |
| 1. Topographical comparison between the study and the test phase lifetime exposure effect in the LPC window (p. 8) | anteriority × laterality × phase: F (1, 42) = 7.86, p = .008, $\hat{\eta}_{G}^{2}$ = .007 | Nonsignificant anteriority × laterality × phase: F (1, 42) = 2.73, p = .11, $\hat{\eta}_{G}^{2}$ = .002  However, there is a significant anteriority x phase interaction: F (1, 42) = 10.68, p = .002, $\hat{\eta}_{G}^{2}$ = .028 |  |
| 1. ANOVA on the (F)N400 amplitude in the test phase (p. 10) | Marginally significant anteriority × response interaction: F (1,46) = 4.04, p = .050, $\hat{\eta}_{G}^{2}$ < .001  Significant 4-way interaction of anteriority × laterality × task × response: F (1, 46) = 7.08, p = .011, $\hat{\eta}_{G}^{2}$ < .001 | Nonsignificant anteriority × response interaction: F (1,46) = 2.88, p = .097, $\hat{\eta}_{G}^{2}$ < .001  Significant 4-way interaction of anteriority × laterality × task × response: F (1, 46) = 10.11, p = .003, $\hat{\eta}_{G}^{2}$ < .001 | 453 ms to 460 ms, on electrode Fp1, significant task × response: T_WJt_/c (1.0,42.0) = 10.74, p = .002.  Post hoc test showed that high lifetime responses elicited more positive voltage: T_WJt_/c (1.0,42.0) = 7.98, p = .008. (figure S1f) |
| 1. (F)N400 topographical comparison between the two tasks in the test phase (p. 10) | anteriority × laterality × task, F (1, 46) = 5.73, p = .021, $\hat{\eta}_{G}^{2}$ = .003 | Nonsignificant anteriority × laterality × task, F (1, 46) = 1.88, p = .18, $\hat{\eta}_{G}^{2}$ = .002  However, there is a marginally significant interaction of laterality x task: F (1, 46) = 3.80, p = .057, $\hat{\eta}_{G}^{2}$ = .005 |  |
| 1. Study phase frequency effect in the (F)N400 time window (p. 10) | anteriority × presentation frequency interaction, F (1, 47) = 4.45, p = .040, $\hat{\eta}_{G}^{2}$ = .001 | Nonsignificant anteriority × presentation frequency interaction, F (1, 47) = 2.87, p = .097, $\hat{\eta}_{G}^{2}$ = .001 | No factor resembling an (F)N400 was identified. |
| 1. Topographical comparison between the study and the test phase frequency effect in the (F)N400 window (p. 10) | anteriority × laterality × phase, F (1, 42) = 8.23, p = .006, $\hat{\eta}_{G}^{2}$ = .005 | Nonsignificant, all ps > .13 |  |
| 1. Comparing (F)N400 lifetime exposure effect between the two tasks in the test phase (p. 11) | no significant interactions involving lifetime exposure × task, all ps > .08 | Significant lifetime exposure x task interaction, F (1, 46) = 5.96, p = .019, $\hat{\eta}_{G}^{2}$ = .009  Post-hoc t-tests showed that the effect of lifetime exposure was numerically more consistent with the polarity of the (F)N400 in the lifetime exposure task, t (46) = 1.60, p = .058, d = 0.23 | No factor resembling an (F)N400 was identified. |
| 1. Lifetime exposure effect in the study phase in the (F)N400 time window (p. 11) | anteriority × laterality × normative lifetime exposure, F (1, 42) = 6.61, p = .014, $\hat{\eta}_{G}^{2}$ < .001 | anteriority × laterality × normative lifetime exposure, F (1, 42) = 5.46, p = .024, $\hat{\eta}_{G}^{2}$ < .001 | No factor resembling an (F)N400 was identified. |
| 1. Topographical comparison between the study and the test phase lifetime exposure effect in the (F)N400 window (p. 11) | No significant interaction involving the factor “experimental phase”, all ps > .3 | No significant interaction involving the factor “experimental phase”, all ps > .5 |  |


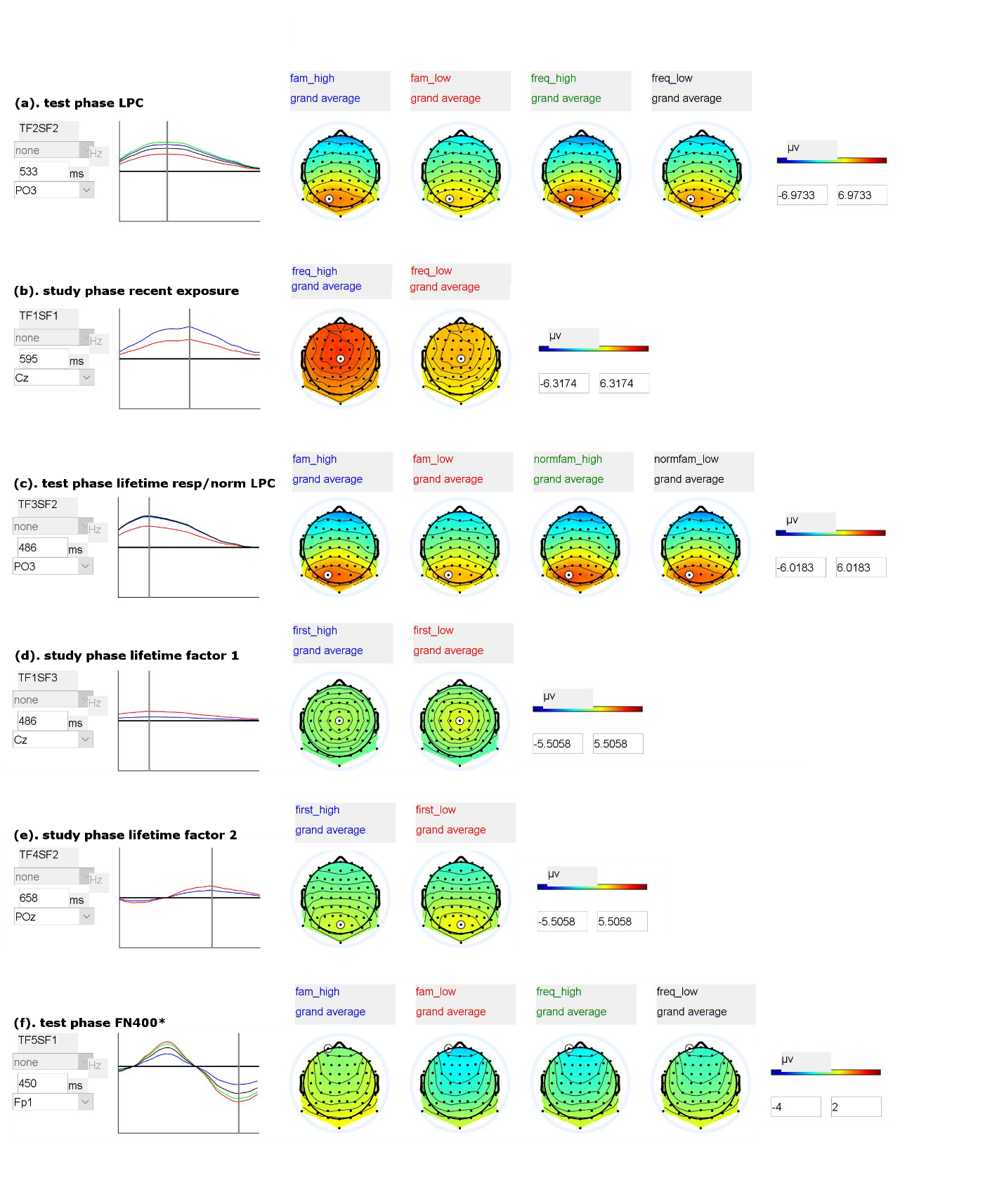


*Figure S1. (a)-(e) components with a positive peak in LPC time window, (f) a component with a negative peak in FN400/N400 time window. Principle components largely replicated the effects acquired in analyses using mean ERP amplitude. The figure shows peak latencies and electrodes detected through the autoPCA process, along with the waveform of the principal components and corresponding topographies in each condition. Please refer to Table S1 for more contrast details for each subpanel.*

References

Dien, J. (2010). The ERP PCA Toolkit: An open source program for advanced statistical analysis of event-related potential data. *Journal of Neuroscience Methods*, *187*(1), 138–145. https://doi.org/10.1016/j.jneumeth.2009.12.009

Dien, J. (2012). Applying Principal Components Analysis to Event-Related Potentials: A Tutorial. *Developmental Neuropsychology*, *37*(6), 497–517. https://doi.org/10.1080/87565641.2012.697503
